# Implications of tumor-positive sentinel lymph nodes in single vs multiple nodal basins in melanoma

**DOI:** 10.3389/fonc.2024.1416685

**Published:** 2024-07-08

**Authors:** Emiliano Reyes, Kristel Lourdault, Judi Anne Ramiscal, Stacey Stern, Richard Essner

**Affiliations:** ^1^ Melanoma and Cutaneous Oncology Lab, Borstein Family Melanoma Program, Saint John’s Cancer Institute, Santa Monica, CA, United States; ^2^ Department of Surgical Oncology, Saint John’s Cancer Institute, Santa Monica, CA, United States; ^3^ Data Management/Biostatistics, Saint John’s Cancer Institute, Santa Monica, CA, United States

**Keywords:** tumor-positive lymph node, nodal basin, survival, prognosis, staging, melanoma

## Abstract

**Background:**

Melanoma patients’ prognosis is based on the primary tumor characteristics and the tumor status of the regional lymph nodes. The advent of lymphoscintigraphy with SLN biopsy (SLNB) has shown that melanoma can drain to multiple nodal basins but the significance of multiple basins (vs. one basin) with tumor-positive sentinel lymph node(s) (+SLN) of similar tumor burden has not been shown. We examined the impact of the number of nodal basins with +SLN (+basin) in melanoma patients and its significance for patients’ prognosis and survival.

**Study design:**

We identified 1,915 patients with +SLN from two randomized surgical clinical trials: Multicenter Selective Lymphadenectomy Trials I and II. Patient groups were divided based on number of +SLNs and number of +basins. Disease-free survival (DFS), distant disease-free survival (DDFS) and melanoma-specific survival (MSS) were compared with the Kaplan-Meier method and log-rank tests. Univariable and multivariable analyses were performed using Cox proportional hazard regressions.

**Results:**

Among the 1,915 patients, 1,501 had only one +SLN (78%) in one basin and 414 (22%) had multiple +SLNs: 340 located in one basin and 74 in multiple basins. Among patients with multiple +SLNs, those with multiple +basins have a worse DFS, DDFS and MSS than those with a single basin (p ≤ 0.0001 for all comparisons). MSS was significantly different based on AJCC stages: AJCC IIIA and IIIB (p ≤ 0.001 and 0.0287, respectively).

**Conclusion:**

Our results suggest that the number of tumor-positive basins may be important for staging and in understanding the biology of lymph node metastases.

## Introduction

The prognosis of melanoma patients is directly linked to the Breslow thickness and ulceration status of the primary tumor, as well as the tumor status of the regional lymph nodes (1–5). In accordance with the American Joint Committee on Cancer (AJCC) classification system (8^th^ edition), patients found to have tumor-positive sentinel lymph node(s) (+SLN) are categorized as stage III which is divided into four subgroups (A to D) based on the number of +SLNs, the Breslow thickness and ulceration status of the primary tumor. Melanoma specific survival (MSS) for AJCC stage III patients decreases as their subgroup increases. The 5-year MSS ranges from 93% to 32% for AJCC stages IIIA to IIID patients (2).

When the disease spreads to distant organs, patients are classified as AJCC stage IV. Without systemic therapies, AJCC stage IV patients have an even worse overall survival (OS) than stage III patients with: 28%, 10.7% and less than 10% at 1-, 2 and years, respectively (6, 7). The site of metastases impacts patients’ survival; patients with lung metastases tend to have a better OS than patients with brain metastases (6, 8). Additionally, the number of metastatic sites affects patients’ OS. Blach et al., has shown that the 1-year OS for patients with a single metastatic site is better than patients with 2 or 3 sites (36% compared to 13% and 0%, respectively) (9). This observation made us wonder if the number of nodal basins with +SLN (+basin) could also impact patients’ survival.

With the advent of lymphoscintigraphy, the standard of care for melanoma patients has drastically changed. Elective lymph node dissection (ELND) was the standard approach for lymph node staging until Morton’s work that described SLNB - a minimally invasive approach to replace traditional ELND (10). The use of lymphoscintigraphy led to a better understanding of lymphatic drainage and the discovery of potential drainage to multiple lymph node basins for melanoma patients. Howard et al., demonstrated that lymphatic drainage to multiple basins was not prognostically significant but the study didn’t examine the importance of tumor burden in simultaneous basins due to the lack of lymphoscintigraphy in their study and the use of traditional ELND (11). Thus, we decided to evaluate the importance of the number of basins with +SLN – one vs multiple - on melanoma patients’ survival.

## Materials and methods

### Patient population

This study includes data from two prospectively collected randomized surgical trials: Multicenter Selective Lymphadenectomy Trial-I and -II (MSLT-I and -II) (12, 13). Based on the clinical trial design, none of the enrolled patients had clinically distant or in-transit disease prior to enrollment. The databases were queried to identify patients diagnosed between 1994 and 2014, with at least one +SLN detected from SLNB. +SLN were determined by Hematoxylin and Eosin and Immunohistochemistry staining that was standardized for each study (14). The cohort was divided based on the number of +SLNs found (1 vs. >1) and the number of basins with +SLN (1 vs. >1). After the surgical treatment (see MSLT-I and -II guidelines) of their primary tumor and SLNB, patients were monitored by clinical exam, blood test, and radiographic imaging to detect recurrences. Recurrence was defined as development of metastases of any type: local, in-transit, regional, or distant. For the MSLT-I trial, the follow-up schedule was every 3 months for years 1 and 2, every 4 months during year 3, every 6 months for years 4 and 5, then annually until year 10. For the MSLT-II trial, the follow-up schedule was every 4 months for years 1 and 2, every 6 months for years 3 and 4 and then annually until year 10. Compliance for patient follow-up schedule (i.e., the number of patients who survived or died within the time period) were 81.9% (1569/1915 patients) at 5 years and 42% (806 of 1915 patients) at 10 years. Patients’ clinical and pathological data are summarized in **Table 1**.

**Table 1:** Patients’ clinical and pathological characteristics.

	Total		1+LN 1+Basin		>1+LN 1+Basin		>1+LN >1+Basin		p-value
	1915		1501	78.40%	340	17.70%	74	3.90%	
Gender									0.2291
Female	798	41.70%	627	41.80%	147	43.20%	24	32.40%	
Male	1117	58.30%	874	58.20%	193	56.80%	50	67.60%	
Age									0.3048
<60	1275	66.60%	999	66.60%	221	65.00%	55	74.30%	
≥ 60	640	33.40%	502	33.40%	119	35.00%	19	25.70%	
Primary Site									<.0001
Extremity	798	41.70%	623	41.50%	165	48.50%	10	13.50%	
Head/Neck	198	10.30%	158	10.50%	30	8.80%	10	13.50%	
Trunk	919	48.00%	720	48.00%	145	42.60%	54	73.00%	
Breslow									0.0107
≤1mm	174	9.10%	143	9.50%	22	6.50%	9	12.20%	
1.1-2.0mm	674	35.20%	547	36.40%	109	32.10%	18	24.30%	
2.1-4.0mm	700	36.50%	544	36.20%	131	38.50%	25	33.80%	
>4mm	367	19.20%	267	17.80%	78	22.90%	22	29.70%	
Ulceration									0.0314
Absent	1142	59.60%	919	61.20%	181	53.20%	42	56.80%	
Present	754	39.40%	568	37.80%	156	45.90%	30	40.50%	
Unknown	19	1.00%	14	1.00%	3	0.90%	2	2.70%	
number of +SLN(s)									Not applicable
1	1501	78.38%	1501	100.00%	0	0.00%	0	0.00%	
2	355	18.54%	0	0.00%	294	86.47%	61	82.43%	
3	40	2.09%	0	0.00%	30	8.82%	10	13.51%	
>3	19	0.99%	0	0.00%	16	4.71%	3	4.05%	
number of basin with +SLN(s)									Not applicable
1	1841	96.10%	1501	100.00%	340	100.00%	0	0.00%	
2	74	20.70%	0	0.00%		100.00%	74	100.00%	
number of +NSN									<.0001
No	1886	98.50%	1499	99.90%	313	92.10%	74	100.00%	
Yes	29	1.50%	2	0.10%	27	7.90%	0	0.00%	
CLND									0.9108
No	927	48.40%	728	48.50%	165	48.50%	34	46.00%	
Yes	988	51.60%	773	51.50%	175	51.50%	40	54.00%	
Path stage									<.0001
IIIA	680	35.50%	556	37.00%	102	30.00%	22	29.70%	
IIIB	514	26.80%	418	27.80%	81	23.80%	15	20.30%	
IIIC	716	37.40%	527	35.10%	153	45.00%	36	48.60%	
IIID	5	0.30%	0	0%	4	1.20%	1	1.40%	

### Statistical analysis

Demographic and clinicopathologic factors were compared between the three groups using the Chi-square test or, when applicable, the Fisher’s Exact test. Disease-Free Survival (DFS), Distant Disease-Free Survival (DDFS) and Melanoma Specific Survival (MSS) curves were generated using the Kaplan-Meier method with p-values from the log-rank test. DFS, DDFS and MSS compared the 3 groups (one +SLN in one basin, multiple +SLNs in one basin and multiple +SLNs in multiple basins).

Multivariable proportional hazard analyses were performed to identify factors that contribute to DFS, DDFS and MSS. We utilized SAS software, version 9.4 (SAS Institute) for all analyses. All tests were two-sided, and statistical significance was set at p<0.05.

## Results

### Patient characteristics

This study consisted of 1,915 patients who underwent SLNB and had at least 1 +SLN (**Table 1**). Most patients were under the age of 60 years old (n=1,275; 66.6%) and male (n=1,117; 58.3%). Primary tumors were located mostly on the trunk (n=919; 48.0%), followed by the extremities (n=798; 41.7%) and the head/neck region (n=198; 10.3%). Most primary tumors were not ulcerated (n=1,142; 59.6%). 71.7% of patients (n=1,374) had Breslow thickness between 1.1mm-4.0mm. The majority of patients had only one +SLN (n=1,501; 78.4%) localized in one basin; the remaining 414 patients had multiple +SLNs in either one basin (n=340; 17.7%) or multiple basins (n=74; 3.9%). Patients with multiple +SLNs had usually two +SLNs (n=355, 18.5%). Few patients had tumor-positive non sentinel lymph nodes (+NSN) (n=29; 1.5%) discovered during SLNB. Half of the patients had complete lymph node dissection (CLND) based on MSLT I & II protocols: 51.5% (773/1501) for patients with one +SLN in one basin, 51.5% (175/340) for patients with multiple +SLNs in one basin and 54.0% (40/74) for patients with multiple +SLNs in multiple basins.

Of the 1915 patients, 305 (15.9%) received adjuvant interferon alfa (FDA approved in 1996), 215 (11.2%) received immune checkpoint inhibitors (i.e., ipilimumab, nivolumab, pembrolizumab; all from MSLT-II patients) and 135 (7%) received BRAF/MEK therapy (all from MSLT-II) as a treatment in the course of advanced disease.

Trunk primaries were the most common for patients with multiple +SLNs in multiple basins (n=54; 73.0%). Patients who had +SLNs in multiple basins typically had thicker melanoma (>4mm) (29.7%) compared to those patients with only one tumor-positive basin: 17.8% for one +SLN and 22.9% for multiple +SLNs in one basin.

### DFS, DDFS and MSS multivariable analysis

The factors associated with increased risk of recurrence were: male gender (HR 1.18, 95% CI 1.02–1.36), increasing age ≥ 60 (HR 1.33, 95% CI 1.16–1.53), increasing Breslow thickness (1.1–2.0mm: HR 1.85, 95% CI 1.27–2.68; 2.1–4.0mm: HR 2.86, 95% CI 1.66–4.94; >4.0mm: HR 3.74, 95% CI 2.01–6.96), presence of ulceration (HR 2.01, 95% CI 1.55–2.61), the number of +SLNs and number of tumor-positive basins (multiple +SLNs in one basin: HR 1.44, 95% CI 1.22–1.70 and multiple +SLNs in multiple basins: HR 1.46, 95% CI 1.07–1.98) (**Supplementary Table 1**). Meanwhile, multivariable analyses showed that having a CLND (HR 0.80, 95% CI 0.70–0.92) after +SLN was associated with improved DFS.

The risk of distant disease (DDFS) increased with: male gender (HR 1.21, 95% CI 1.03–1.42), increasing age ≥ 60 (HR 1.33, 95% CI 1.14–1.56), increasing Breslow thickness (2.1–4.0mm: HR 1.98, 95% CI 1.09–3.60, >4.0mm: HR 2.87, 95% CI 1.45–5.68), presence of ulceration of the primary tumor (HR 1.84, 95% CI 1.39–2.44), increasing number of +SLNs and increasing number of tumor positive basins (multiple +SLNs in one basin: HR 1.35, 95% CI 1.12–1.63) and multiple +SLNs in multiple basins: HR 1.61, 95% CI 1.16–2.22) (**Supplementary Table 2**).

Finally, male gender (HR 1.29, 95% CI 1.06–1.56), increasing age ≥60 (HR 1.23, 95% CI 1.03–1.48), increasing Breslow Thickness (1.1–2.0mm: HR 1.85, 95% CI 1.08–3.18, 2.1–4.0mm: HR 2.63, 95% CI 1.25–5.53, >4.0mm: HR 3.99, 95% CI 1.75–9.10), presence of ulceration of the primary tumor (HR 2.13, 95% CI 1.53–2.95), increasing number of +SLNs and number of basins (multiple +SLNs in one basin: HR 1.34, 95% CI 1.07–1.67, multiple +SLNs in multiple basins: HR 1.69, 95% CI 1.16–2.44) were associated with increased risk of melanoma specific death (**Table 2**).

**Table 2:** MSS, Univariable and multivariable analysis.

Parameters	Univariable			Multivariable		
	HR	95% CI	p-value	HR	95% CI	p-value
Gender						
Female (ref)	1			1		
Male	1.48	1.23-1.77	<001	1.29	1.06-1.56	0.01
Age						
<60 (ref)	1			1		
≥60	1.34	1.12-1.61	0.001	1.23	1.03-1.48	0.024
Primary Site						
Extremity (ref)	1			1		
Head/Neck	1.22	0.91-1.64	0.183	1.18	0.87-1.60	0.288
Trunk	1.17	0.97-1.41	0.092	1.12	0.92-1.35	0.266
Breslow, mm						
≤1.0 (ref)	1			1		
1.1-2.0	2.11	1.26-3.56	0.005	1.85	1.08-3.18	0.025
2.1-4.0	3.75	2.25-6.23	<.001	2.63	1.25-5.53	0.011
>4.0	6.16	3.68-10.3	<.001	3.99	1.75-9.10	0.001
Ulceration						
Absent (ref)	1			1		
Present	2.65	2.22-3.16	<.001	2.13	1.53-2.95	<.001
Unknown	2.57	1.27-5.21	0.009	1.8	0.87-3.72	0.111
+NSN						
No (ref)	1			1		
Yes	1.48	0.81-2.68	0.202	1.01	0.54-1.89	0.97
CLND						
No (ref)	1			1		
Yes	1.15	0.96-1.37	0.122	1.13	0.94-1.35	0.19
Path stage						
IIIA (ref)	1			1		
IIIB	1.91	1.46-2.50	<.001	1,03	0.64-1.66	0.906
IIIC	3.68	2.92-4.65	<.001	0.96	0.44-2.09	0.916
IIID	13	4.78-35.5	<,001	1.76	0.46-6.73	0.411
LN/Basin Group						
1+LN 1+basin (ref)	1			1		
>1+LN, 1+basin	1.5	1.22-1.86	<,001	1.34	1.07-1.67	0.01
>1+ LN, >1+basin	2.13	1.49-3.05	<.001	1.69	1.16-2.44	0.006

### DFS, DDFS and MSS

**Figure 1:**
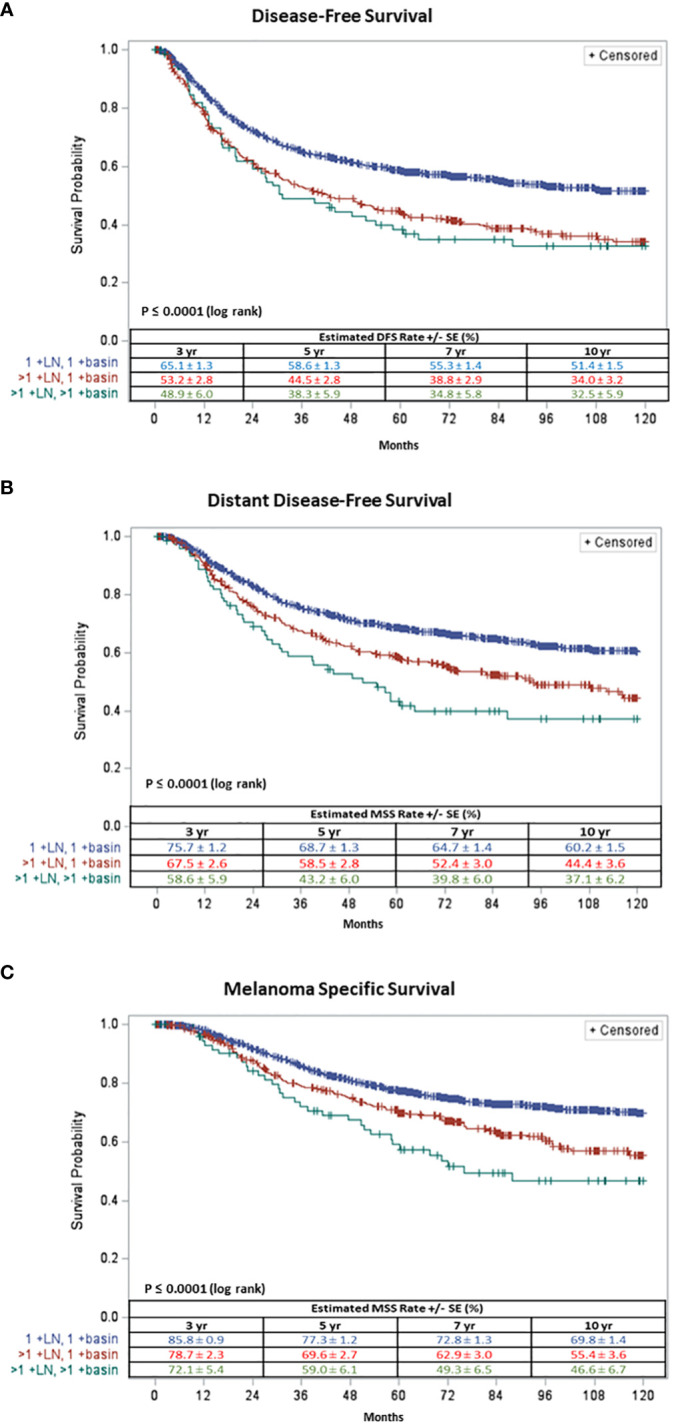
Ten-year **(A)** Disease Free-Survival and **(B)** Distant Disease Free-Survival and **(C)** Melanoma Specific Survival of patients with one +SLN in one basin (in blue), multiple + SLNs in one basin (in red) and multiple +SLNs in multiple basins (in green).

Patients with one +SLN in one basin had a significantly better 5-year DFS (58.6% ± 1.3) than those with multiple +SLNs in one basin (44.5% ± 2.8) and those with multiple +SLNs in multiple basins (38.3% ± 5.9) (log rank p<.0001) (**Figure 1A**).

Similarly, patients with one +SLN in one basin had a significantly better 5-year DDFS (68.7% ± 1.3) compared to patients with multiple +SLNs in one basin (58.5% ± 2.8) and those with multiple +SLNs in multiple basins (43.2% ± 6.0) (log rank p<.0001) (**Figure 1B**).

Patients with one +SLN in one basin demonstrated a significantly better 5-year MSS (77.3% ± 1.2) compared to those with multiple +SLNs in one basin (69.6% ± 2.7) and those with multiple +SLNs in multiple basins (59.0% ± 6.1) (log rank p<.0001) (**Figure 1C**).

### Survival based on the primary tumor site: trunk, head/neck, and extremities

Because most patients with +SLNs in multiple basins had primary tumors located on the trunk (**Table 1**), we assessed if the site of primary tumor would impact MSS. We examined patients who had a primary located on the trunk (n=919); 720 (78.3%) had one +SLN in one basin; 145 (15.8%) had multiple +SLNs in one basin and 54 (5.9%) had multiple +SLNs in multiple basins. The 5-year MSS was 75.0% ± 1.7, 73.1% ± 4.0 and 64.4% ± 7.0 for patients with one +SLN in one basin, those with multiple +SLNs in one basin and those with multiple +SLNs in multiple basins (log rank p=0.0085), respectively (**Supplementary Figure 1A**).

We evaluated patients with primary tumors on the extremities (n=798); 623 (78.1%) had one +SLN in one basin; 165 (20.7%) had multiple +SLNs in one basin and 10 (1.2%) had multiple +SLNs in multiple basins. The 5-year MSS was 80.7% ± 1.7, 67.4% ± 3.9 and 48.0% ± 16.5 for patients with one +SLN in one basin, those with multiple +SLNs in one basin and those with multiple +SLNs in multiple basins (log rank p=0.0001), respectively (**Supplementary Figure 1B**).

Finally, we examined the data of patients with primary tumors in the head/neck region (n=198); 158 (79.8%) had one +SLN in one basin; 30 (15.2%) had multiple +SLNs in one basin and 10 (5.1%) had those with multiple +SLNs in those with multiple basins. The 5-year MSS was 73.9% ± 3.8, 65.6% ± 8.8 and 44.4% ± 16.6 for patients with one +SLN in one basin, those with multiple +SLNs in one basin and those with multiple +SLNs in multiple basins (log rank p=0. 1783), respectively (**Supplementary Figure 1C**).

### Survival based on the patient’s AJCC stage

**Figure 2:**
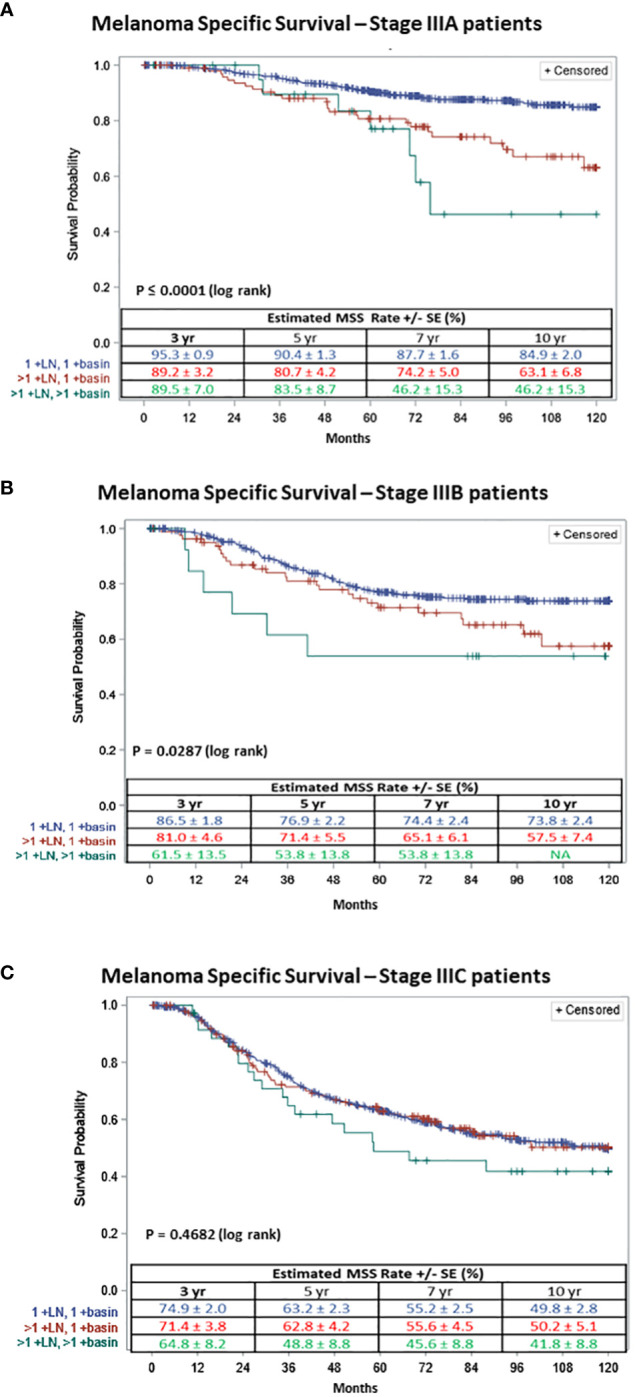
Ten-year Melanoma Specific Survival of patients with **(A)** AJCC stage IIIA, **(B)** AJCC stage IIIB or **(C)** AJCC stage IIIC; with one +SLN in 1 basin (in blue), multiple +SLNs in 1 basin (in red) and multiple +SLNs in multiple basins (in green).

It is known that an increase in AJCC stage correlates with decrease in survival, thus we sought to assess the impact of the number of +SLNs and basins in patients with similar AJCC stages, i.e., stage IIIA, IIIB and IIIC. We compared the MSS among the three groups: one +SLN in one basin, multiple +SLNs in one basin and multiple +SLNs in multiple basins (**Figure 2**).

For patients with AJCC stage IIIA melanoma (n=680), 556 (81.8%) had one +SLN in one basin, 102 (15.0%) had multiple +SLNs in one basin and 22 (3.2%) had multiple +SLNs in multiple basins. The 5-year MSS for stage IIIA patients was 90.4% ± 1.3 for one +SLN in one basin, 80.7% ± 4.2 for multiple +SLNs in one basin and 83.5% ± 8.7 for multiple +SLNs in multiple basins. The survival curves diverge even more at 10 years (log rank p≤ 0.0001) (**Figure 2A**).

For patients with AJCC stage IIIB melanoma (n=514), 418 (81.3%) had one +SLN in one basin, 81 (15.8%) had multiple +SLNs in one basin and 15 (2.9%) had multiple +SLNs in multiple basins. The 5-year MSS for stage IIIB patients was 76.9% ± 2.2, 71.4% ± 5.5 and 53.8% ± 13.8 for patients with one +SLN in one basin, those with multiple +SLNs in one basin and those with multiple +SLNs in multiple basins (log rank p≤0.0287) (**Figure 2B**), respectively.

Finally, for AJCC stage IIIC melanoma patients (n=716), 527 (73.6%) had one +SLN in one basin, 153 (21.4%) had multiple +SLNs in one basin and 36 (5.0%) had multiple +SLNs in multiple basins. The 5-year MSS for stage IIIC patients was 63.2% ± 2.3, 62.8% ± 4.2 and 48.8% ± 8.8 for patients with one +SLN in one basin, patients with multiple +SLNs in one basin and those with multiple +SLNs in multiple basins (log rank p≤0.4682) (**Figure 2C**), respectively.

## Discussion

A number of studies have demonstrated that the 5-year MSS rate ranges from 93% to 32% for patients with +SLNs compared to 99% to 82% for those without +SLN, due to increasing risk of recurrence for patients with increasing number of +SLNs (15–19). Approximately 2/3 of all AJCC stage III melanoma patients will succumb to their disease within 5 years of diagnosis (20, 21). Thus, it is crucial for patients’ staging and treatment that those with regional lymph node disease are identified. MSLT-I established regional LN staging using SLNB with pre-operative lymphoscintigraphy that now serves as the standard of care for melanoma patients. Indeed, the number of tumor-positive lymph nodes, independent of their sentinel status, is the most important criterion for staging and prognosis. However, the number of basins with +SLNs is not included in the AJCC staging system.

The aim of our study was to investigate the importance of having +SLNs in multiple basins as we hypothesized that there may be an impact on patients’ survival, and to determine if this criterion should potentially be included in the staging system and the decision to initiate systemic therapy. We compared patients’ outcomes between three groups based on their number of +SLNs and basins: one +SLN in one basin, multiple +SLNs in one basin and multiple +SLNs in multiple basins, and demonstrated that patients with multiple +SLNs in multiple basins had significantly worse DFS, DDFS and MSS when compared to those with +SLN(s) in only one basin (one +SLN in one basin and multiple +SLNs in one basin), independent of the number of +SLNs. These findings align with previous reports that the survival of patients with +SLNs in multiple basins was found to be worse when compared to those with +SLN(s) in a single basin, regardless of the number of +SLNs involved. It is also reported that patients with multiple +SLNs have worse survival than those with single +SLN activity regardless of the number of basins involved (13, 22). Multivariable analyses confirmed that the number of +SLNs and the number of +basins is associated with worse DFS, DDFS and MSS.

Patients with primary tumors located on the trunk or the extremities yielded similar results: patients with multiple +SLNs had worse MSS than those with one +SLN and that patients with +SLNs in multiple basins also had worse MSS than those with +SLNs in one basin. Similarly, AJCC stage IIIA and IIIB patients with multiple +SLNs in multiple basins had worse 5-year MSS than the other two groups. These results suggest that the number of basins is important for MSS and should potentially be included in the staging system.

Our study has limitations by its retrospective nature and by the limited number of patients in certain groups, such as patients with +SLNs in multiple basins. Indeed, only a very small portion of patients with +SLN had multiple basins (3.9%). There is a need for a larger cohort to validate our findings.

Our results showed that both having multiple +SLNs compared to a single +SLN and having +SLNs in multiple basins compared to one basin are associated with a worse survival. Currently, the AJCC staging system does not make any distinction between patients with single or multiple positive nodal basin involvement; it only considers the total number of +SLNs. Based on our results, we believe that the number of basins with +SLNs should be included in the staging system.

## Author’s note

Presented in part at Society of Surgical Oncology Annual Meeting, oral presentation, March 19^th^, 2021.

## Data availability statement

The data analyzed in this study is subject to the following licenses/restrictions: Data is available upon request to the corresponding autho. Requests to access these datasets should be directed to richard.essner@providence.org.

## Ethics statement

The studies involving humans were approved by Providence Health and Services and eIRB protocol studies 2020000522 and 2019000139. The studies were conducted in accordance with the local legislation and institutional requirements. Written informed consent for participation was not required from the participants or the participants’ legal guardians/next of kin in accordance with the national legislation and institutional requirements. Written informed consent was obtained from the individual(s) for the publication of any potentially identifiable images or data included in this article.

## Author contributions

ER: Visualization, Methodology, Writing – original draft, Writing – review & editing, Formal analysis, Data curation. KL: Writing – original draft, Writing – review & editing, Visualization, Supervision, Project administration, Methodology, Formal analysis, Data curation. JR: Writing – original draft, Writing – review & editing, Methodology, Formal analysis, Conceptualization. SS: Writing – original draft, Writing – review & editing, Visualization, Methodology, Formal analysis, Conceptualization. RE: Writing – original draft, Writing – review & editing, Supervision, Methodology, Funding acquisition, Formal analysis, Conceptualization.

## Supplementary material

Supplementary Figure 1 Ten-year Melanoma Specific Survival of patients with primary tumor in **(A)** trunk, **(B)** extremity or **(C)** head and neck region; with one +SLN in one basin (in blue), multiple +SLNs in 1 basin (in red) and multiple +SLNs in multiple basins (in green).
