# Supplementary figures and images for: Implications of tumor-positive sentinel lymph nodes in single vs multiple nodal basins in melanoma
Source: Front Oncol. 2024 Jul 8;14:1416685. doi: 10.3389/fonc.2024.1416685 (PMC11260672; doi:10.3389/fonc.2024.1416685)

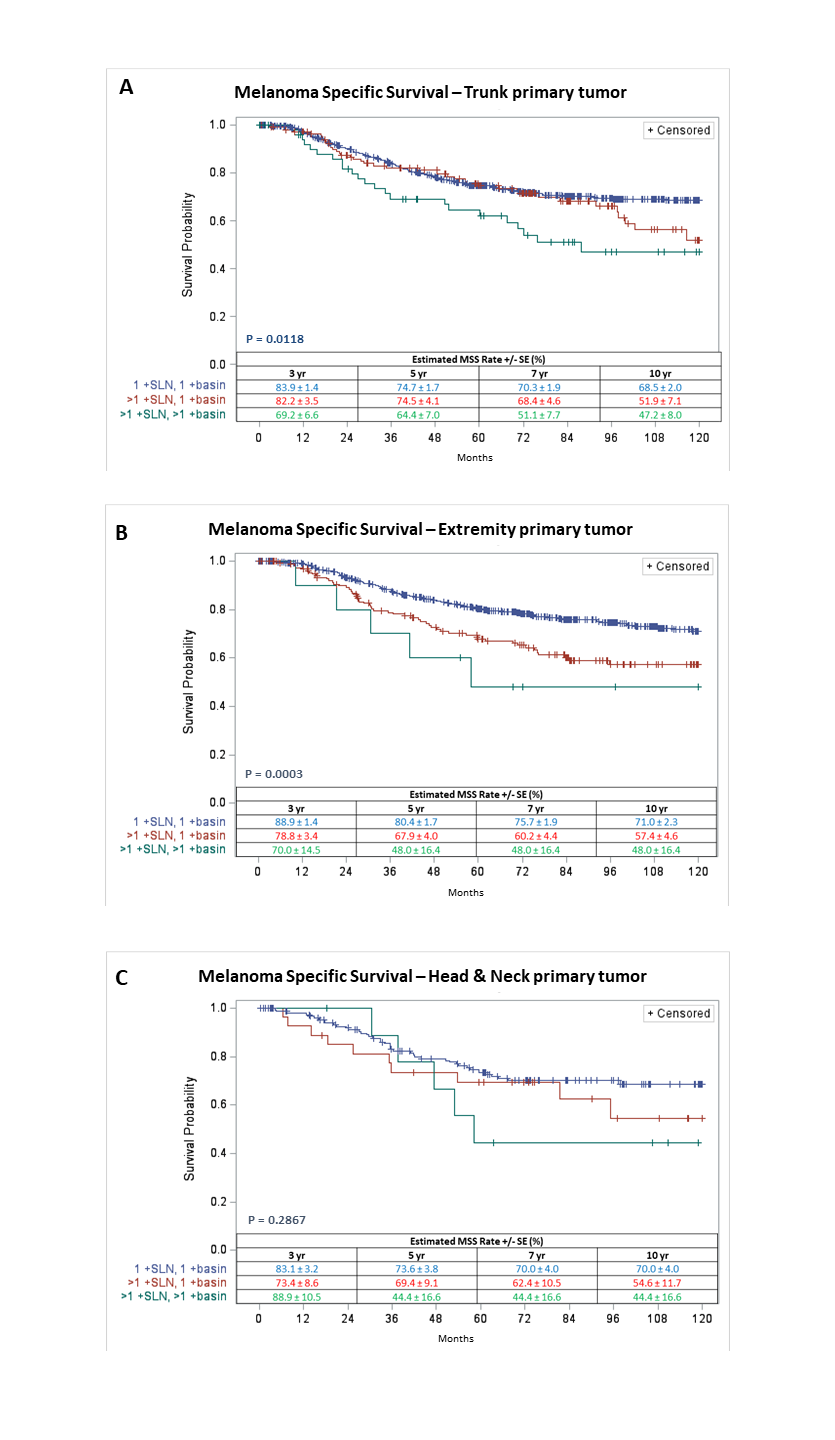

Supplement: Supplementary Figure 1 — Ten-year Melanoma Specific Survival of patients with primary tumor in (A) trunk, (B) extremity or (C) head and neck region; with one +SLN in one basin (in blue), multiple +SLNs in 1 basin (in red) and multiple +SLNs in multiple basins (in green). [file Image_1.tif]
